# Does maxillomandibular fixation affect skeletal stability following mandibular advancement? A single-blind clinical trial

**DOI:** 10.1186/s40902-022-00350-w

**Published:** 2022-05-06

**Authors:** Reza Tabrizi, Arash Sarrafzadeh, Shervin Shafiei, Hamidreza Moslemi, Ramtin Dastgir

**Affiliations:** 1 Department of Oral and Maxillofacial Surgery, School of Dentistry, Shahid Beheshti University of Medical Sciences, Danshjoo BLVD, Velenjak, Shahid Chamran Highway, Tehran, Iran; 2 Department of Oral and Maxillofacial Surgery, School of Dentistry, Arak University of Medical Sciences, Arak, Iran; 3 Faculty of Dentistry, Tehran Medical Sciences, Islamic Azad University, Tehran, Iran

**Keywords:** Orthognathic surgery, Skeletal class II malocclusion, Bilateral sagittal split osteotomy

## Abstract

**Background:**

The stability of the results remains a significant concern in orthognathic surgeries. This study aimed to assess the amount of relapse following mandibular advancement with/without maxillomandibular fixation (MMF).

**Materials and methods:**

A single-blind clinical trial was conducted on patients with mandibular retrognathism who underwent BSSO for mandibular advancement and Lefort I maxillary superior repositioning. Patients were randomly divided into two groups of treatment (MMF) and control (no MMF). In the treatment group, MMF was performed for 2 weeks; meanwhile, MMF was not performed in the control group, and only guiding elastics were applied postoperatively. Lateral cephalograms were obtained preoperatively (T1), immediately after surgery (T2), and at 1 year postoperatively (T3). The distance from points *A* and *B* to the *X* and *Y* plane were measured to identify the amount of vertical and horizontal relapse in 1 year as a primary outcome. An independent *t*-test was applied in order to find differences in outcomes between the control and treatment groups.

**Results:**

Fifty-eight patients were evaluated in two groups (28 patients in the MMF group and 30 in the no-MMF group). The magnitude of mandibular advancement following BSSO was 7.68±1.39 mm and 7.53±1.28, respectively, without significant difference among the groups (*p*= 0.68). The mean sagittal and vertical changes (relapse) at point *B* were significantly different between the two groups at 1-year follow-up after the osteotomy (*p*=0.001 and *p*=0.05, respectively).

**Conclusion:**

According to the results of this study, patients with short-term MMF following BSSO for mandibular advancement benefit from significantly greater skeletal stability in the sagittal and vertical dimensions.

## Background

Skeletal class II malocclusion is a common dentofacial deformity that is often associated with esthetic, functional, and psychological problems. Moderate to severe mandibular retrognathism often requires combined orthodontic and surgical treatments to bring about optimal functional and esthetic results. Bilateral sagittal split osteotomy (BSSO) is the most common approach to correct mandibular retrognathism via orthognathic surgery [1, 2]. Despite the overall satisfaction of patients and surgeons with the results of BSSO, short-term and long-term stability of the results remains a significant concern [3]. Considerable amounts of research have been conducted to investigate the effect of surgical variables such as different surgical approaches and fixation methods on the occurrence of postoperative relapse [4]. Some relevant recent studies have also mentioned the effects of some non-surgical confounding factors such as the mandibular plane (MP) angle, the magnitude of advancement, and counterclockwise rotation on the extent of post-surgical relapse [2, 3, 5–7].

Other contributing factors may include postoperative care and modalities, such as functional therapy, elastic therapy, or maxillomandibular fixation (MMF). In cases of mandibular advancement of more than 6 to 8 mm, mainly when it is associated with advancement genioplasty, early relapse often occurs in the first 6 to 8 weeks postoperatively due to the rotation and slippage of the osteotomy site. Furthermore, substantial advancements result in chronic compression loading on the condyles. This compression force could cause a relapse in the long-term due to the condylar remodeling. Epker recommended a 3-week MMF period for stabilization of the osteotomy segments [8]. However, the use of supplemental MMF to increase the stability of the results following mandibular advancement is challenging.

This study’s purpose is to address the following question: Among the patients who have undergone sagittal osteotomy for mandibular advancement, does MMF affect the stability of surgical results? We hypothesized that MMF would decrease the rate of relapse following mandibular advancement. Therefore, the present study aimed to assess the occurrence of skeletal relapse in sagittal and vertical dimensions following mandibular advancement with/without MMF.

## Materials and methods

The investigators designed and implemented a single-blind clinical trial. The sample was derived from the population of patients introduced to the Oral and Maxillofacial Surgery Department of Shahid Beheshti University of Medical Sciences between January 1, 2016, and December 31, 2019. The study protocol was approved by the committee of the medical ethics group of Shahid Beheshti University of Medical Sciences (IR.SBMU.DRC.REC.1398.101) and registered in the Iranian Registry of Clinical Trials (IRCT201911260445510N1). Patients with following conditions were enrolled in the present study: (I) patients with class II skeletal deformity who underwent BSSO for mandibular advancement by 5 to 10 mm and conventional Le Fort I osteotomy, (II) patients who attended all the follow-up sessions, and (III) availability of complete patient records including their preoperative and postoperative lateral cephalograms. Subjects were excluded from the study enrollment if they had an asymmetry, a history of previous surgery, a history of trauma to the mandible, bad split during their surgical operation, a surgical bone augmentation, temporomandibular joint disorders, temporomandibular joint surgery, genioplasty, or craniofacial syndromes such as palatal cleft; failed to return for follow-up; or refused study enrollment.

Patients were randomly divided into two groups of treatment and control. An independent researcher made random allocation cards using computer-generated random numbers. Then, the cards were placed in sealed envelopes.

Age, gender, the mean of mandibular and maxillary movements in the vertical and horizontal directions, OC plane, and MP changes were variables of the study. The relapse at the *A* and *B* points in the vertical and horizontal directions were outcomes of the study. The use of MMF was considered as a predictor factor.

### Surgical method

A medial short cut osteotomy was performed for all patients, and lateral osteotomy was carried out based on the method described by Obwegeser [9] and modified by Dal Pont [10]. Internal fixation was performed bilaterally with a 2.0-mm semi-rigid miniplate and four monocortical screws. Conventional Le Fort I osteotomy with no rotation was performed in order to correct the maxillary position. The maxilla was placed in an ideal position and fixed using 4 L-shaped 1.8 mm titanium non-locking miniplates with four mini-screws (6 mm) for each plate. All surgical procedures were performed by one oral and maxillofacial surgeon. In the treatment group, MMF was performed for 2 weeks, and after the release of MMF, guiding elastics were applied for the following 6 weeks. In the control group, MMF was not implemented and only guiding elastics were applied for 8 weeks postoperatively. All patients were referred to their orthodontist 2 months after surgery. MMF was performed with placement of 4 MMF screws (with a 2-mm diameter and 12-mm length): one screw was placed between the canine and the first premolar of the maxilla and mandible on each side.

### Radiographic assessments

**Fig. 1:**
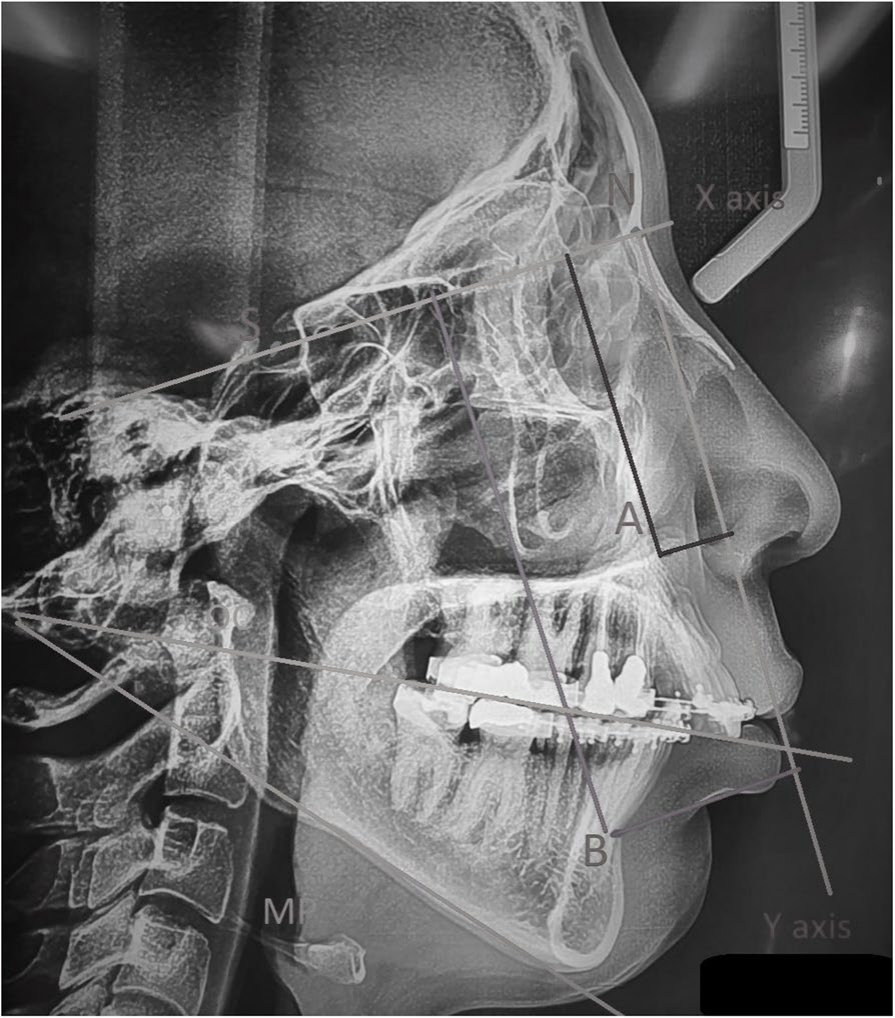
Landmarks on lateral cephalograms

We used lateral cephalograms to evaluate the changes in patients (Fig. 1). The radiographs were taken preoperatively (T1), immediately after surgery (T2), and at 1 year postoperatively (T3). All radiographs were taken in standard natural head position using the same equipment and optimal exposure settings.

### Tracing technique

Two examiners who were blinded to the group allocations of the patients studied the cephalograms. All cephalograms were traced using Dolphin Imaging Version 8.0 (Dolphin Imaging, Chatsworth, CA) following the identification of cephalometric landmarks (Table 1, Fig. 1). The distances from the points *A* and *B* to the *X* and *Y* lines were measured to identify vertical and horizontal relapse. The OP angle was used to determine any maxillary impaction or mandibular rotation in the sagittal plane. The OP angle was defined as the angle between the OP and the SN line. The MP and OP angles were measured before and after surgery to confirm the uniformity of the two groups pre- and postoperatively.

**Table 1:** Landmarks used for tracing of lateral cephalograms

Landmark	Definition
**Point** ***A*** **(subspinale)**	The most posterior point in the concavity between ANS and prosthion
**Point** ***B*** **(supramentale)**	The most posterior midline point in the concavity of the mandible between the most superior point on the alveolar bone overlying the mandibular incisors and pog
**Me (Menton)**	The most inferior point on the symphysis
**Pog (pogonion)**	The most anterior point on the symphysis
**ANS (anterior nasal spine)**	Anterior tip of the sharp bony process of the maxilla
**PNS (posterior nasal spine)**	Posterior spine of palatal bone
**Mandibular plane**	Line tangent to inferior border of the mandible
**S (sella)**	Geometric center of pituitary fossa
**N (nasion)**	Most anterior point on frontonasal suture
**SN**	Line connecting sella and nasion
***X*** **line**	SN line
***Y*** **line**	Line crossing the *S* point perpendicular to X line

### Statistical analysis

The statistical analyses were performed using SPSS version 23 (SPSS Inc., IL, USA).

Age, gender, preoperative mandibular plane angle (pre-op MP), postoperative occlusal plane angle (post-op OP), postoperative mandibular plane angle (post-op MP), immediate postoperative maxillary vertical movement (immediate post-op *A*-*Y* subtracted from pre-op *A*-*Y*), immediate postoperative maxillary horizontal movement (immediate post-op *A*-*X* subtracted from pre-op *A*-*X*), immediate postoperative mandibular vertical movement (immediate post-op *B*-*Y* subtracted from pre-op *B*-*Y*), and immediate postoperative maxillary horizontal movement (immediate post-op *B*-*X* subtracted from pre-op *B*-*X*) were the primary variables in the present study.

The normal distribution of primary continuous variables was confirmed by the Skewness and Kurtosis analysis. The chi-square test was applied to analyze gender distribution in the two groups. The outcome of the present study was a skeletal relapse in vertical and horizontal dimensions which were evaluated by vertical change at point *A* at 1 year after the operation (1-year post-op *A*-*Y* subtracted from immediate post-op *A*-*Y*), horizontal change at point *A* at 1 year after the operation (1-year post-op *A*-*X* subtracted from immediate post-op *A*-*X*), vertical change at point *B* at 1 year after the operation (1-year post-op *B*-*Y* subtracted from immediate post-op *B*-*Y*), and horizontal change at point *B* at 1 year after the operation (1-year post-op *B*-*X* subtracted from immediate post-op *B*-*X*). An independent *t*-test was utilized in order to find the differences in primary variables and study outcomes between the two groups. Statistical significance was set at *p*<0.05. Inter-rater reliability analysis was performed using the Kappa statistics to assess the inter-examiner agreement.

## Results

Initially, 60 subjects were included in the study (30 subjects in each group). Two subjects were removed from the study since they did not return for their follow-up. Eventually, 58 subjects were studied in two groups (28 patients in the treatment group and 30 in the control group). Immediate sagittal advancement relapse with signs such as acute temporomandibular disorders (TMD), malocclusion, hardware failure, immediate overjet increase, and deviation was not observed among the subjects.

The distribution of males and females was not significantly different in the two groups (*p*=0.58, Table 2). The mean age of patients was 24.43±3.78 years old in the treatment group and 24.60±4.61 years old in the control group. There was no difference in the mean age between the two groups (*p*=0.88). The mean MP angle was 29.14±2.35° and 30.03±1.96°, respectively, with no statistically significant difference (*p*=0.12). The mean change in the MP angle immediately after surgery was 5.39±1.73° in the treatment group and 5.20±1.32° in the control group. No significant difference was observed between the two groups in this respect (*p*=0.63). The mean change in the OP angle was not significantly different between the two groups (*p*=0.27).

**Table 2:** Mean amount of immediate changes in the treatment (MMF) and control (non-MMF) groups

Variables	MMF group	No-MMF group	***P***-value	95% CI	
**Gender**	10 males, 18 females	11 males, 19 females	0.58*	-	
**Age (years)**	24.43±3.78	24.60±4.61	0.88**	−2.40	2.05
**Preoperative MP**	29.14±2.35	30.03±1.96	0.12**	−2.02	0.24
**Preoperative OC plane**	10.75±1.04	10.73±1.14	0.95**	−0.56	0.59
**Postoperative MP change**	5.39±1.73	5.20±1.32	0.63**	−0.61	0.99
**Postoperative OP change**	4.86±1.41	5.27±1.41	0.27**	−1.15	0.33
**Vertical change at point** ***B*** **immediately after the operation (mm)**	7.68±1.39	7.53±1.28	0.68**	−5.56	0.84
**Horizontal change at point** ***B*** **immediately after the operation (mm)**	5.07±1.25	5.13±0.97	0.83**	−0.64	0.52
**Vertical change at point** ***A*** **(mm) immediately after the operation**	1.21±0.50	1.43±0.57	0.12**	−0.50	0.63
**Horizontal Change at** **point** ***A*** **(mm) immediately after the operation**	5.46±1.75	5.26±1.02	0.62**	−3.19	1.59

*Chi-square test

**Independent *t*-test

*CI* confidence interval, *MP* mandibular plane, *OP* occlusal plane

The mean magnitude of mandibular change at point *B* in the vertical dimensions immediately after osteotomy was 7.68±1.39 mm in the MMF group and 7.53±1.28 mm in the no-MMF group. There was no difference in the mean vertical change at point *B* immediately after surgery between the two groups (*p*=0.68). The mean magnitude of horizontal mandibular change at point *B* immediately after osteotomy was 5.07±1.24 mm in the MMF group and 5.13±0.97 mm in the no-MMF group. Analysis of the data did not demonstrate any statistically significant difference for the mean amount of horizontal change at point *B* immediately after surgery between the two groups (*p*=0.83).

The mean magnitude of vertical and horizontal changes of the maxilla at point *A* was not significantly different immediately after the osteotomy between the two groups (*p*=0.12 and *p*=0.62, respectively, Table 2).

The mean magnitude of vertical change at point *B* was 1.07±1.01 mm in the MMF group and 1.93±0.94 mm in the no-MMF group at 1 year after the osteotomy. There was a significant difference in the mean amount of vertical change at point *B* between the two groups (*p*=0.001).

The mean magnitude of horizontal change at point *B* was 1.03±0.69 mm in the MMF group and 1.50±0.51 mm in the no-MMF group at 1 year after the osteotomy. Analysis of the data demonstrated a significant difference in the mean amount of horizontal change at 1 year after the osteotomy between groups 1 and 2 (*p*=0.05).

There were significant differences in the mean amount of vertical and horizontal changes at point *A* at 1 year after the osteotomy (*p*=0.04 and *p*=0.02, respectively; Table 3).

**Table 3:** Mean amount of changes in distances from the points *A* and *B* to the *X* and *Y* lines at 1 year postoperatively in the treatment (MMF) and control (non-MMF)

Outcomes	Group 1	Group 2	***P*** value	95% CI	
Vertical change at point *B* at 1 year postoperation (mm)	1.07±1.01	1.93±0.94	0.001	−1.37	−0.34
Horizontal change at point *B* at 1 year postoperation (mm)	1.03±0.69	1.50±0.51	0.005	−0.78	−0.14
Vertical change at point *A* at 1 year postoperation (mm)	0.61±0.78	1.03±0.76	0.04	−0.83	−0.18
Horizontal change at point *A* at 1 year postoperation (mm)	0.54±0.84	1.10±0.50	0.02	−1.01	−0.11

*CI* confidence interval

The inter-examiner reliability was found to be Kappa=0.62 (*p*<0.001 at 95% CI), which showed a substantial agreement between the examiners.

## Discussion

Post-surgical relapse is an essential concern in the correction of skeletal class II malocclusion via BSSO surgery [1]. The use of supplemental MMF to decrease the relapse rate was the main hypothesis of this study. According to this study’s results, the MMF group had a lower rate of relapse in the vertical and horizontal dimensions at points *A* and *B*. The vertical relapse at point *B* was 14.02% of the initial movement in the MMF group and 25.63% in the non-MMF group. The horizontal relapse at point *B* was 20.31% of the initial movement in the MMF group and 29.23% in the non-MMF group.

According to the study results, the treatment group (MMF group) had a lower rate of relapse compared to the control group (no-MMF) in the mandible and maxilla in 1 year following osteotomies. Chen et al. demonstrated that age, the magnitude of mandibular advancement, preoperative MP, counterclockwise mandibular rotation, and bimaxillary surgery were independent risk factors for long-term sagittal skeletal relapse, whereas preoperative MP, counterclockwise rotation, and the magnitude of mandibular advancement were independent risk factors for vertical relapse [5]. Tabrizi et al. reported that the magnitude of mandibular advancement was a more reliable surgical predictor for horizontal relapse at point *B*. The changes in the MP angle during surgery were responsible for vertical, but not horizontal, relapse at point *B* [3]. According to the study results, the MMF group had a lower rate of relapse in the sagittal and vertical dimensions at points *A* and *B*. The study variables such as age, gender, changes in the MP and OP angles, and the magnitude of maxillomandibular movements were compared between the two groups. The statistical similarity was observed between the two groups for the aforementioned variables, and their effects could be ruled out.

Early relapse seems to be more related to surgical techniques and errors in rigid internal fixation or may even occur when the condyles are not positioned correctly in their locations. However, late relapse and long-term stability would be the result of the functional imbalance of forces that would lead to condylar resorption [1, 5, 11].

The sagittal relapse may occur due to the soft tissue tension and fixation site and has a correlation with the amount of initial movement [11]. Condylar positioning, suprahyoid muscles, and the pterygomasseteric sling are responsible for the vertical relapse at point *B* [12, 13].

Immediate relapse would present itself with TMD, malocclusion, hardware failure, immediate overjet increase, and deviation. Since all surgical procedures were performed by the same oral and maxillofacial surgeon with the same osteotomy technique and none of the mentioned signs were observed, the operator error and its possible role in the surgical relapse rate were minimized.

Since in this study, subjects underwent bimaxillary osteotomies, the change in maxillary position has an impact on the mandibular position as well as mandibular change at the *B* point. It was demonstrated that 1-mm change in the maxillary position leads to 0.71-mm vertical and 0.21-mm horizontal movements of the mandible when the amount of maxillary impaction was 8 mm or less [14]. The results of the study demonstrated a similar initial movement at the *A* point in the vertical and horizontal directions in the treatment and control groups. The initial maxillary movement in the treatment and control groups was 5.46±1.75 mm and 5.26±1.02 mm, respectively. Therefore, the effect of the superior maxillary repositioning on mandibular autorotation and the position of the *B* point was similar in the treatment and control groups. Regarding the similar maxillary superior positioning in the two groups, the change of the *B* point in the vertical and horizontal directions after 1 year demonstrated a reliable relapse rate in the *A* and *B* points. In view of the fact that the superior maxillary repositioning is a stable procedure in bimaxillary osteotomy [15], the significant relapse was related to the mandibular movement.

Hartlev et al. studied the use of intermaxillary fixation (IMF) in mandibular advancement to decrease the relapse rate. They found no difference in the relapse rate between the skeletal IMF group and the control group without skeletal IMF [16]. On the other hand, Schwartz et al. demonstrated that BSSO in combination with skeletal IMF could be used as an alternative to distraction osteogenesis in large mandibular advancements (>10 mm) with equal stability [17].

MMF reduces the soft tissue tension and increases the stability of fixation devices to overcome muscle tension (suprahyoid and pterygomandibular sling) [18]. The advantage of skeletal MMF over dental IMF is the minimal tooth movement, which decreases the tooth relapse after releasing the fixation [18]. Paunonen et al. [8] reported a skeletal relapse rate as high as 25%, which was significant following BSSO advancement surgery despite insignificant dental alterations. This finding highlights the importance of dental relapse and its compensation by the postoperative skeletal relapse [7].

Internal rigid fixation methods have been used by several authors to achieve maximum short-term and long-term skeletal stability following BSSO. Different types of rigid internal fixation have been used following BSSO, such as miniplates with mono-cortical screws, bicortical screws, and a combination of both referred to as the hybrid technique. Most of the published studies have demonstrated minimal differences among these types of internal fixation methods in terms of skeletal relapse; hence, this type of fixation seems to be promising in providing long-term stability [1, 2, 4]. It could be expected that similar MMF’s effects would be achieved by other fixation modalities of mandibular advancement sagittal split-like positional screws.

The disadvantages of MMF include delay in return to normal function and difficulties maintaining proper oral hygiene [14]. Generally, patients are not satisfied with MMF. The use of MMF screws is associated with increased risk of tooth root injury, soft tissue burying of screw heads in the anterior mandibular vestibule, and interference of wire loops with canine facettes or the upper incisor edges [19]. Meanwhile, the clinical difference among groups was 1 mm in the vertical dimension (about 12% of the adsorption rate) in point *B* and 0.5 in the horizontal dimension (about 12% in the amount of change), so it could be recommended; however, the clinical judgment of the practitioner should justify the use of this method. This study was conducted in subjects who needed bimaxillary surgery. Consequently, it was difficult to estimate the relapse rate in the maxilla and mandible independently. For evaluation of MMF’s effect on the relapse of pure mandibular advancement, research on retrognathic patients needing a mandibular advancement is mandatory.

## Conclusion

According to the results of this study, patients with short-term MMF following BSSO for mandibular advancement benefit from significantly higher skeletal stability in the sagittal and vertical dimensions.

## Data Availability

All data generated or analyzed during this study are included in this published article.
